# A Novel Fault Diagnosis System on Polymer Insulation of Power Transformers Based on 3-stage GA–SA–SVM OFC Selection and ABC–SVM Classifier

**DOI:** 10.3390/polym10101096

**Published:** 2018-10-03

**Authors:** Xiaoge Huang, Yiyi Zhang, Jiefeng Liu, Hanbo Zheng, Ke Wang

**Affiliations:** 1 Guangxi Key Laboratory of Power System Optimization and Energy Technology, Guangxi University, Nanning 530004, China; nnhuangxg@163.com (X.H.); yiyizhang@gxu.edu.cn (Y.Z.); 2 National Demonstration Center for Experimental Electrical Engineering Education, Guangxi University, Nanning 530004, China; 3 China Electric Power Research Institute, Haidian District, Beijing 100192, China; wangke1@epri.sgcc.com.cn

**Keywords:** artificial bee colony (ABC), dissolved gas analysis (DGA), fault diagnosis, genetic algorithm (GA), power transformers, simulated annealing (SA) algorithm, support vector machine (SVM)

## Abstract

Dissolved gas analysis (DGA) has been widely used in various scenarios of power transformers’ online monitoring and diagnoses. However, the diagnostic accuracy of traditional DGA methods still leaves much room for improvement. In this context, numerous new DGA diagnostic models that combine artificial intelligence with traditional methods have emerged. In this paper, a new DGA artificial intelligent diagnostic system is proposed. There are two modules that make up the diagnosis system. The two modules are the optimal feature combination (OFC) selection module based on 3-stage GA–SA–SVM and the ABC–SVM fault diagnosis module. The diagnosis system has been completely realized and embodied in its outstanding performances in diagnostic accuracy, reliability, and efficiency. Comparing the result with other artificial intelligence diagnostic methods, the new diagnostic system proposed in this paper performed superiorly.

## 1. Introduction

### 1.1. Motivation

Transformers are distributed in almost all domains of the entire electrical network, changing the values of AC voltage (current) at given points to another or several values without altering the frequency. They not only guarantee the normal operation of the power grid, but also affect people’s living environment [1]. However, the operating conditions of the transformer (including temperature and electromagnetic conditions) are harsh and not conducive to its long-term health [2,3,4]. In the meantime, the failure of power transformers is often attended by disastrous consequences, which include equipment burning and large-scale blackouts. Undoubtedly, the operational safety of power transformers deserves serious concern.

Fault diagnosis is regarded as one of the most important considerations in maintaining the safe operation of the power transformer. Diagnostic and fault prognosis techniques have been widely and successfully applied in numerous engineering dynamic systems [5], and are also of extreme importance to researchers of electrical energy systems [6]. At present, fault diagnosis systems play a critical role in maintaining the operational safety of power transformers, and their principles and designs are constantly updated and strengthened [7,8].

We argue that a sound transformer diagnostic method should be strengthened in the following aspects: (1) Economic efficiency and (2) solving the allowable-time problem. Economic efficiency is related to diagnostic costs. The average annual failure rate of transformers is not very high; it usually does not exceed 5% [9]. In other words, the expense of the transformer fault diagnosis is of minor significance in most cases. When the transformer is operating properly, the diagnosis provides less valuable guidance to maintenance staff. However, the traditional diagnosis costs for the transformer are relatively prominent, due to the lack of online diagnostic methods. Transformers need to be shut down periodically for maintenance and, during such shutdowns, the outage cost of the transformer is huge. Therefore, it is necessary to control the diagnostic costs and improve the economic efficiency of the transformer’s diagnostic method. On the other hand, the allowable-time problem is a noticeable challenge to traditional transformer diagnosis. The fault of the transformer has no obvious abnormality at the beginning, making the allowable-time for maintenance actions relatively short. Under these circumstances, this paper aims to propose an online-diagnosis method that is economical and capable of overcoming the allowable-time problem.

### 1.2. Related Work

Mainstream transformers’ fault diagnostic methods include chemical quantity based methods and electrical quantity based methods [10]. Chemical based methods typically include dissolved gas analysis (DGA) [11], degree of polymerization (DP) measurements [12], moisture analysis (MA) [13], and Furan analysis by high performance liquid chromatography (HPLC) [14], among others. The electrical based methods involve the time domain method [15] and frequency domain polarization measurement [16]. Among these, DGA is the most widely exploited [17]. Since DGA was proposed in 1973, this online method has been widely accepted and exploited all around the world, owing to its outstanding economic efficiency and capability to detect failure in advance, which effectively alleviates the pressure brought on by the allowable-time problem [18]. The DGA works via detecting hydrogen (H_2_), methane (CH_4_), acetylene (C_2_H_2_), ethylene (C_2_H_4_), ethane (C_2_H_6_), carbon monoxide (CO), and carbon dioxide (CO_2_) gases dissolved in the transformer oil, which is produced by pyrolysis of insulation paper (board) cellulose. In this proposal, we divided DGA methods into traditional methods and intelligence methods. Traditional methods include: The Doernerburg Ratio Method [19], Rogers Ratio [20], IEC 60,599 Method [21,22], Duval Triangles Method [23], and Pentagon Method [24]. Despite having a long history, most of these methods are unstable in a complex operating environment. On the other hand, while research of intelligent DGA diagnostic methods has appeared, these are implemented less frequently compared with the traditional methods [25]. Therefore, this paper hopes to improve this situation as much as possible.

The development of an intelligent diagnosis of the power transformer is promising. In general, intelligent diagnosis designs are built on the ideas of traditional methods. They combine most of the advantages of both traditional ideas and intelligent algorithms. Recently, intelligent methods, such as fuzzy logic inference systems [26], artificial neural networks [27], support vector machines (SVM) [28,29], and some other machine learning algorithms have been applied to transformer fault diagnosis and have had impressive performances [10,30,31]. However, limitations also exist with intelligent diagnostic methods. For example, fuzzy inference depends excessively on the experience of researchers [32]. In addition, “local minima” and “overfit” are two of the marked weaknesses of Artificial Neural Network (ANN) [33]. Compared to these methods, the application of SVM in abnormal detection and fault diagnosis has marked advantages [34]. It overcomes the local minimum, dimension, and over-fitting problems, and requires less in the scale of the training sample.

### 1.3. Contribution and Paper Orgnization

**Figure 1:**
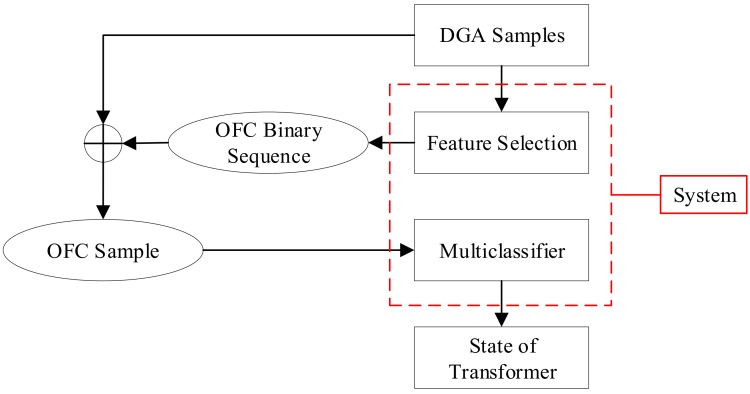
The structure of the diagnostic system.

In this paper, we combined SVM with traditional DGA and proposed a two-stage SVM diagnostic system, and the main structure of the system was depicted in Figure 1. The system contains a feature selection module, which would extract the optimal feature combination (OFC) from DGA information, and a multiclassifier, judging the type of fault in power transformers based on the OFC. Accounting for the relatively low request of the speed and the highlighted accuracy and reliability, a 3-stage GA–SA–SVM selection model which combined a genetic algorithm (GA) [35] and simulated annealing (SA) algorithm [36] with SVM was utilized to complete the selection of OFC. In addition to these, the artificial bee colony (ABC) algorithm [37], which has the fastest iteration speed and the highest search efficiency, was exploited in the diagnostic stage. The entire system has been fully realized, with the accuracy of its result reaching 92%.

The remaining sections of this paper are structured as follows: In Section 2, a 3-stage GA–SA–SVM method, which was used to determine the optimal feature combination of DGA feature sets, is proposed. The ABC–SVM based diagnostic model is constructed in Section 3. A case study of the entire system is illustrated in Section 4. Finally, we conclude the research and identify the direction of future research in Section 5.

## 2. Optimal Feature Combination Selection

### 2.1. The Candidate DGA Feature Sets

In recent years, the DGA gas ratio is used as the characteristic parameter, such as in the Doernerburg Ratio Method [19] and the Rogers Ratio Method [20]. Inspired by [38], eight categories of gas: H_2_, CO, CO_2_, CH_4_, C_2_H_2_, C_2_H_4_, C_2_H_6_, and the total hydrocarbon (TH, TH = CH_4_ + C_2_H_2_ + C_2_H_4_ + C_2_H_6_) were taken into account in this paper. Therefore, there were 28 DGA candidate ratios in total, which are shown in Table 1 below.

### 2.2. DGA Feature Selection Model

**Figure 2:**
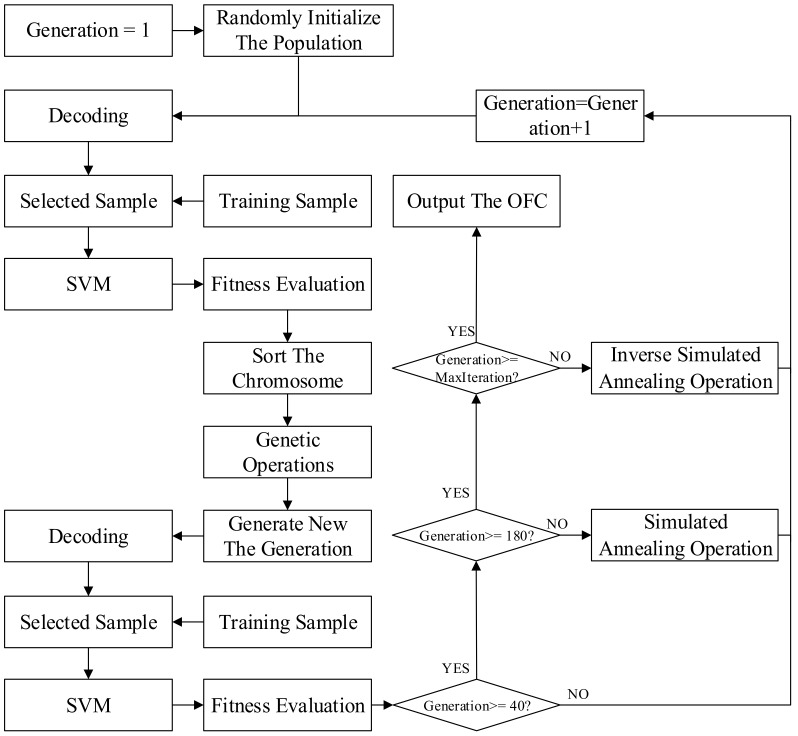
Flowchart of optimization selection based on 3-stage GA–SA–SVM.

The DGA feature selection is necessary due to the ambiguous relationship between the DGA features and the types of transformer fault. According to [39], parts of DGA gas ratios are unrelated to the fault diagnosis, which means that blindly selecting DGA features or even focusing on all features is unwise. To address this problem, it is necessary to select the key DGA features carefully, which was mostly suitable in the following diagnosis. Accounting for the low request of the speed and the highlighted accuracy and reliability, a 3-stage GA–SA–SVM selection model was constructed to complete the selection. The flowchart of the selection process is illustrated in Figure 2, and a brief description of the 3-stage GA–SA–SVM model constructing process is as follows:

#### 2.2.1. Multiclass Nonlinear SVM Model

**Figure 3:**
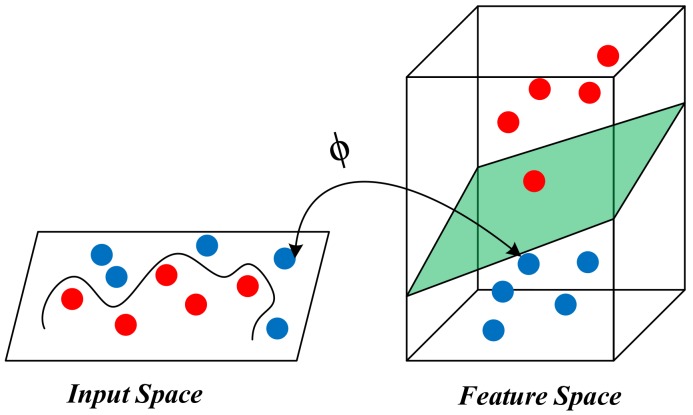
The model of support vector machine (SVM).

The principle of SVM is to find the optimal hyperplane which satisfies the classification requirements and extends the distance of the two data sets on the hyperplane as much as possible. The model of the SVM is depicted in Figure 3.

Assuming that $x_{i}\in R_{n}$ and $y_{i}\in R_{n}$ are the input and output of the training set, respectively, the training set {(*x*_1_, *y*_1_), …, (*x_i_*, *y_i_*), …, (*x_l_*, *y_l_*)} was obtained. At the same time, a class table *y_i_* ∈ {−1, +1} was introduced, which was determined through the given *x_i_*. The constraints of the training set data can be written as (1):

(1) $$\begin{array}{l} \omega^{T}\varphi (x_{i})+b\leq -1,\;\mathrm{if}\;y_{i}=-1\\ \omega^{T}\varphi (x_{i})+b\geq +1,\;\mathrm{if}\;y_{i}=+1 \end{array}$$

where the *φ*(*x_i_*) is a nonlinear mapping. Both the *φ*(*x_i_*) and *ω* contain infinite dimensions. They form the optimal hyperplane together.

When the data are linearly inseparable, a non-negative slack variable *ξ_i_* is introduced to transform the SVM into (2):

(2) $$\begin{array}{l} \min\Phi (\omega ,\xi )=\frac{1}{2}{\left\|\omega \right\|}^{2}+C\sum_{i=1}^{l}\xi_{i}\\ s.t.\left\{ \begin{array}{l} y_{i}\left(\omega^{T}x_{i}+b\right)\geq 1-\xi_{i}\\ \xi_{i}\geq 0,i=1,2,\ldots ,l \end{array}\right. \end{array}$$

where parameter *C* is the penalty factor. *C* was determined through optimization, which depends on the GA and SA algorithms.

Build a Lagrangian function to solve the QP problem of (2):

(3) $$\begin{array}{l} L(\omega ,b,\xi ,\alpha ,\beta )=\Phi (\omega ,\xi )\\ -\sum_{i=1}^{l}\alpha_{i}\{y_{i}[\omega^{T}\varphi (x_{i})+b]-1+\xi_{i}\}-\sum_{i=1}^{l}\beta_{i}\xi_{i} \end{array}$$

Among them, *α_i_* > 0 and *β_i_* > 0 are Lagrange multipliers; then, the original problem has been transformed into a quadratic programming problem:

(4) $$\max\Psi (\alpha )=-\frac{1}{2}\sum_{i=1}^{l}\alpha_{i}\alpha_{j}y_{i}y_{j}K(x_{i},x_{j})+\sum_{i=1}^{l}\alpha_{i}$$

(5) $$\sum_{i=1}^{l}\alpha_{i}y_{i}=0,\;\alpha_{i}\in [0,C],\;i=1,\ldots ,l$$

where *K*(*x_i_*, *x_j_*) is called the kernel function that satisfies (6). *σ* was a given parameter, which was determined through the GA and SA optimization.

(6) $$K(x_{i},x_{j})=\exp\left(-\frac{x_{i}-x_{j}}{\sigma^{2}}\right)$$

Using the One-Against-One (OAO) method to extend the two-class SVM to a multiclass SVM, the optimization problem translated into (7):

(7) $$\begin{array}{l} \min\Phi (\omega ,\xi )=\frac{1}{2}{(}^{\omega^{jk})T}\omega^{jk}+C\sum_{i=1}^{l}\xi_{i}^{jk}\\ s.t.\left\{ \begin{array}{l} {(}^{\omega^{jk})T}\varphi (x_{i})+b^{jk}\geq 1-\xi_{i}^{jk},\;y_{i}=j\\ {(}^{\omega^{jk})T}\varphi (x_{i})+b^{jk}\leq \xi_{i}^{jk}-1,\;y_{i}=k\\ \xi_{i}^{jk}\geq 0,\;i-1,2,\ldots ,l \end{array}\right. \end{array}$$

Therefore, the expression of the decision function is written as (8):

(8) $$f^{jk}(x)=sign[{\left(\omega^{jk}\right)}^{T}\varphi (x)+b^{jb}]$$

#### 2.2.2. Application of Genetic Algorithm

Genetic algorithms contributed to the OFC selection for determining *C* and *σ*. They contained three main units: Coding, fitness calculation, and genetic operation.

- Chromosome coding

**Figure 4:**
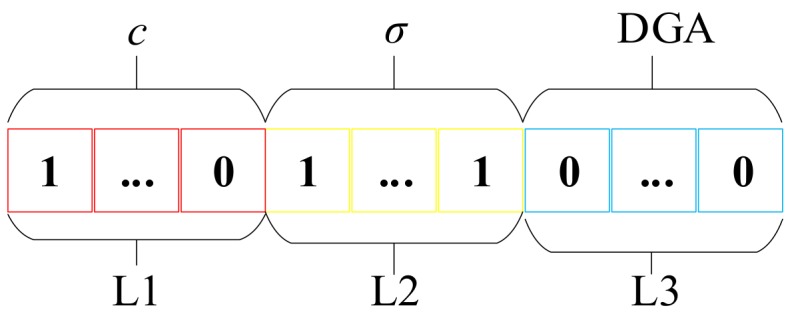
The binary encoding of chromosomes.

The chromosome in GA was abstracted as the solution of the objective function. The abstract process is known as coding. As shown in Figure 4, three sets of parameters: *C*, *σ*, and DGA ratio sets need to be optimized. They match three segments of binary codes which were represented by *L*_1_, *L*_2_, and *L*_3_, respectively. In the first two codes, namely *L*_1_ and *L*_2_, the value of binary encoding in the decimal form is equivalent to the value of its corresponding *C* and *σ*. The binary code on the *L*_3_ segment reflects the combination of selected DGA ratios. The “1” on each bit in *L*_3_ means the corresponding DGA ratio is selected, and “0” reflects the opposite meaning.

- Genetic fitness calculation

Genetic fitness is calculated as follows in (9), which is the standard to evaluate the performance of a single chromosome:

(9) $$f(L_{1},L_{2},L_{3})=-\frac{1}{k}\sum_{i=1}^{k}\left(\frac{l_{T}^{i}}{l^{i}}\times 100\%\right)$$

where, *l^i^* is the number of samples in the *i*th verification set; $l_{T}^{i}$ is the correct classified number in the verification set; and *k* is the number of cross validation. The concept of K-fold cross-classification will be illustrated in the following description.

- Genetic operations

The old solution generates new solutions through genetic operations.

Genetic operation refers to the fact that in each generation, individual chromosomes are chosen according to their selection probability. After that, chromosomes still need to experience crossover and mutation in order to generate a new population. This process ensures that the new population is more adaptable to the environment than the previous generation. The selection probability of each individual is calculated as follows in (10):

(10) $$P_{i}=\frac{f_{i}}{\sum_{i=1}^{N}f_{i}}$$

*f_i_* is the *i*th genetic fitness.

Crossover operations followed (11) with a given certain probability:

(11) $$\begin{array}{l} x_{i}=ax_{i}+(1-a)x_{i+1}\\ x_{i+1}=(1-a)x_{i}+ax_{i+1} \end{array}$$

where *a* is a random number in the interval [0, 1].

The mutation operation (12) refers to randomly selecting a mutation bit *j* in the mutated chromosome and setting it as a normalized random number *U*(*a_i_*, *b_i_*). *a_i_*, *b_i_* are the upper and lower constraints of the corresponding mutation.

(12) $$x_{j}=\left\{ \begin{array}{ll} U(a_{i},b_{i}) & \;if\;\;i=j\\ x_{i} & \mathrm{otherwise} \end{array}\right.$$

#### 2.2.3. Combination of SA Algorithm and GA

**Figure 5:**
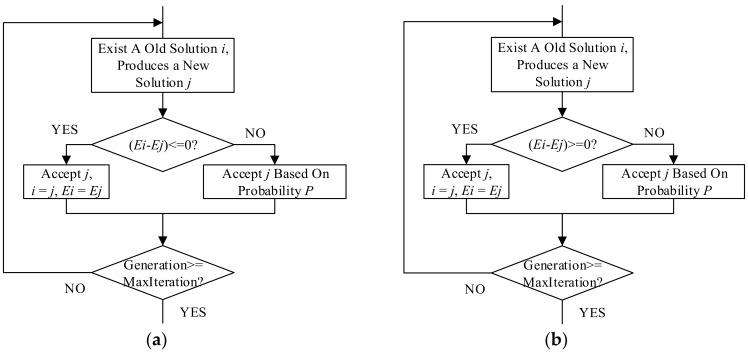
The flowcharts of the simulated annealing operation and the inverse annealing operation. (**a**) The simulated annealing operation; (**b**) The inverse simulated annealing operation.

The SA operation and the inverse SA operation were combined with the GA to acquire a more impressive performance in optimization. The flowcharts of the SA operation and the inverse SA operation are presented in Figure 5.

- Simulated annealing operation

The SA operation is the most noticeable difference between the SA algorithm and other greedy algorithms. The SA operation refers to a reservation principle for the new solution set up in iterations. In the SA operation, the solution of a new generation is retained based on a probability, which is calculated following the Metropolis criterion.

- Metropolis criterion

The SA algorithm draws on the relationship between the temperature *T* and the internal energy *E* in the solid annealing principle. The Metropolis criterion describes the relationship between the probability of accepting a solution of the new generation and *T* and *E*. Assuming that, at temperature *T*, the number of current iterations is *i* and the number of new iterations is *j*, if *E_j_* < *E_i_*, save *j* as the current generation; otherwise, follow the probability *P* to determine whether *j* would be saved, where *P* followed (13). In the SA algorithm, *T* is positively related to the number of iterations, and *E_i_* is numerically equal to the fitness of the *i*th generation.

(13) $$P=\exp\left(-\frac{E_{j}-E_{i}}{KT}\right)$$

- Inverse simulated annealing operation

Inverse simulated annealing operation is the opposite of simulated annealing operation: If *E_j_* > *E_i_*, accept *j* as the current generation; otherwise, follow the probability *P* to accept *j* as the current generation.

- Multi stage of GA–SA-combination

In order to obtain the result of selection with high accuracy, sound stability, and a relatively short time-consumption, we utilize the GA algorithm, the GA hybrid SA (GA–SA) algorithm, and the Inverse SA hybrid GA (Inverse SA–GA) algorithm in multiple stages of generation intervals. Only one of these algorithms runs in each interval to optimize the SVM parameter and OFC.

Through combining GA and SA in multiple stages, we obtained a multistage-GA–SA–SVM selection model.

#### 2.2.4. K-Fold Cross-Validation

K-fold cross-validation (CV) was hired to verify the accuracy of selection. To imply K-fold CV, the initial sample is divided into *K* subsamples, a separate subsample is retained as the data for the validation model, and the other *K* − 1 samples are employed for training. The cross-validation will repeat for *K* times. Each subsample will act as a verified sample once. The average result of validations in *K* times is viewed as the estimation result [29]. Here we set *K* = 5.

## 3. Fault Diagnosis Model Based on ABC–SVM

ABC is a mature algorithm which has been widely applied in solving numerous optimization problems due to its prominent convergence characteristics [37,40]. In order to obtain the highest operating efficiency and the highest diagnostic accuracy, we utilized the ABC algorithm to optimize the SVM parameters, and constructed the ABC–SVM based transformer fault diagnosis model.

### 3.1. The Mechanism of ABC

**Figure 6:**
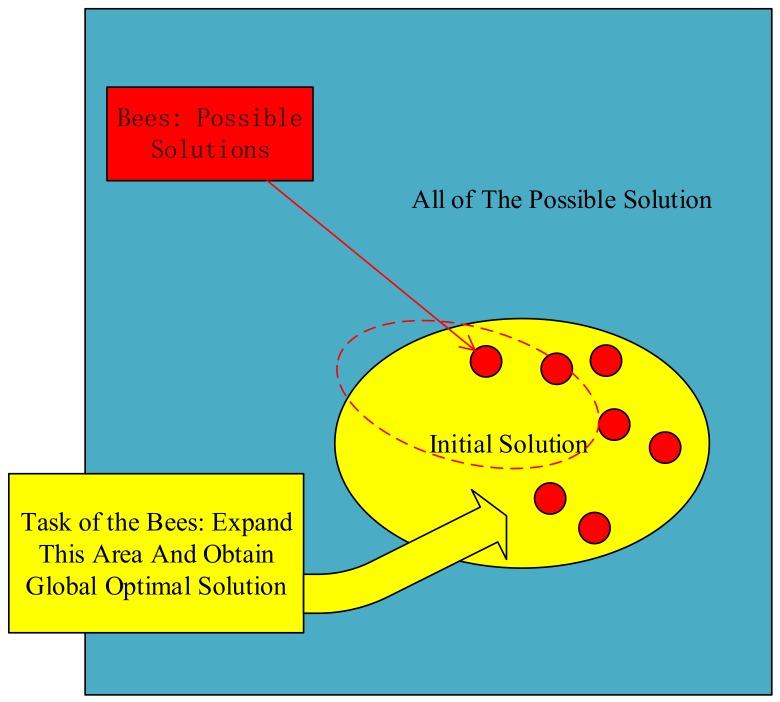
Mechanism of artificial bee colony (ABC).

There are four key components in ABC: Honey sources which abstract into points in the solution space, lead bees, follow bees, and reconnaissance bees. The bees represent the potential solution to the problem. The tasks for each bee are different. As shown in Figure 6, they extend the known honey source to search for the global optimal solution in predetermined manners. Search manners consist of three steps: (1) Lead bees discover a source of the honey and share the source information; (2) each follow bee selects the source to collect the honey according to the information and evaluates the quality of the source; (3) the lead bee converts into a reconnaissance bee and continues to search for new sources near the hive when a source is found repeatedly, but the quality is not improved. When a high-quality source is found, he turns his role back into that of a lead bee. These three steps will be replicated until the best honey source is found.

### 3.2. ABC Optimization Model

In the ABC model, the quality of the honey source *i* (*i* = 1, 2, …, *NP*) corresponds to the fitness value *fit_i_* of the solution, and *NP* is the number of honey sources. The numbers of lead bees and follow bees equal to half of the bee colony, respectively, which also equal the number of honey sources. A honey source accommodates only one bee at any one time.

Let the dimension of the solution problem be *D*. The position of the honey source at the *t*th generation is denoted as $X_{i}^{t}=[x_{i1}^{t},x_{i2}^{t},\ldots ,x_{iD}^{t}]$, where $x_{id}\in (L_{d},U_{d})$, *L_d_* and *U_d_* denote the lower and upper constraints of the search space, respectively, and *d* is a random integer in [1, *D*]. The initial position of the honey source *i* is randomly generated in the search space according to (14).

(14) $$x_{id}=L_{d}+\mathrm{rand}(0,1)\cdot (U_{d}-L_{d})$$

To start the search, the lead bee searches around the honey source *i* according to (15) to generate a new honey source:

(15) $$v_{id}=x_{id}+\varphi (x_{id}-x_{jd})$$

where j∈{1,2,…,NP}, j≠i, this indicates randomly selecting a honey source that is not equal to *i* among the *NP* honey sources; *φ* is a random number of [−1, 1], which is uniformly distributed and determines the magnitude of perturbation. 

Then, the follow bee calculates the fitness of the new honey source $V_{i}=[v_{i1}v_{i2}\ldots v_{id}]$ according to (16) and decides whether or not to replace *X_i_* or keep *X_i_* by using the greedy choice method.

(16) $$fit_{i}=\left\{ \begin{array}{l} 1/(1+f_{i}),f\geq 0\\ 1+\mathrm{abs}(f_{i}),\;\mathrm{otherwise} \end{array}\right.$$

*f_i_* represents the objective function whose functional value is numerically equal to the mean square error (MSE) of the accuracy of the SVM prediction model.

After that, follow bees use the Roulette Wheel Selection to determine the lead bees they follow. The probability in the Roulette Wheel Selection was calculated through (17):

(17) $$P_{i}=\frac{f_{i}}{\sum_{i=1}^{N}f_{i}}$$

**Figure 7:**
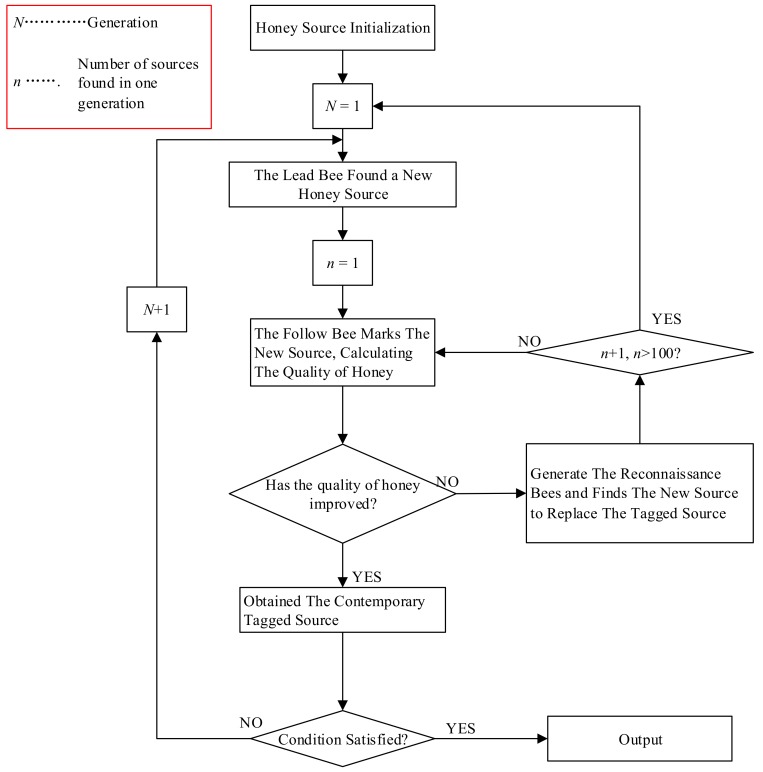
Flowchart of ABC.

During the search process, if a source *X_i_* reaches the limit through trial iterations without finding a better source, the source *X_i_* will be abandoned. The lead bee turns into the role of reconnaissance bee and generates a new source of honey in the search space followed randomly (18). ABC algorithm flowcharts were depicted in Figure 7.

(18) $$X_{i}^{t+1}=\left\{ \begin{array}{l} L_{d}+\mathrm{rand}(0,1)\cdot (U_{d}-l_{d}),t>limit\\ X_{i}^{t},t<limit \end{array}\right.$$

### 3.3. Leave-P-Out Cross Validation

In the diagnostic phase, the LpO CV was exploited for verification. The LpO CV refers to using the *p* elements in the full set *X* as the testing set, and the remaining *n*-*p* elements as the training set. As a result, *p* verification results will be obtained in the end. The final result is numerically equal to the percentage of correct results in *p* results.

### 3.4. Process of Classification Based on ABC–SVM

**Figure 8:**
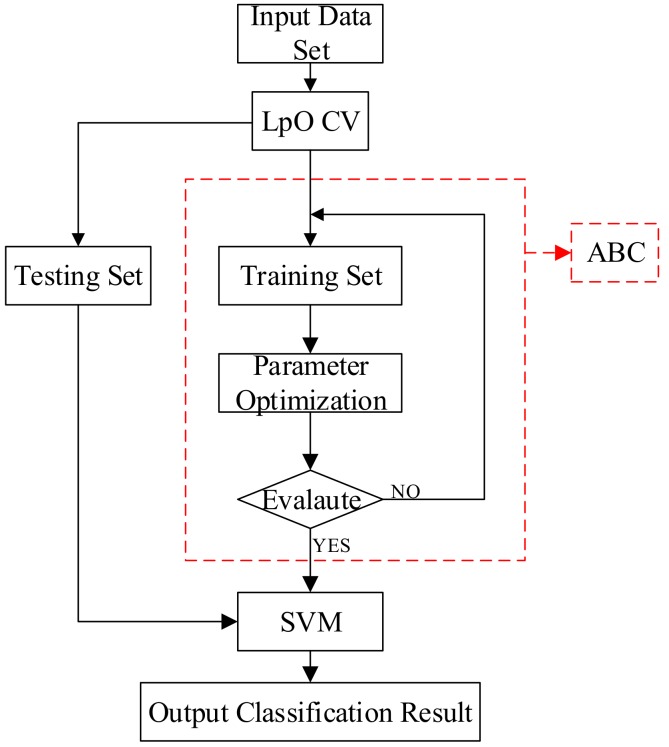
Flowchart of ABC–SVM classification.

The established ABC model is applicable for selecting the optimal parameters of a nonlinear multiclass SVM (*C* and *σ*). The flowchart of ABC–SVM is given in Figure 8, which contains four steps:

Step 1. Utilize LpO CV to generate a training set and a testing set. The training set was sent to the ABC model.
Step 2. Use training set and nonlinear multiclassification support vector machine to construct unknown parameters and to form the optimal objective function.
Step 3. Apply ABC to find the best solution to determine the best parameters of SVM. The best parameters are obtained when the training accuracy meets the threshold of checking; otherwise, step 3 is replayed.
Step 4. Input testing is set to SVM, then the output will be obtained.

## 4. Case Study and Analysis

### 4.1. Data Preprocessin

In this research, 118 sets of transformer fault data which originated from International Electrotechnical Commission Technical Committees (IEC TC) 10 databases [20] were engaged to carry out the test. We labeled the 118 datasets with five states of transformers, which contained: 23 sets of low-energy discharge (LED, represented by “1”), 45 sets of high-energy discharge (HED, represented by “2”), 10 sets of low and middle-temperature overheat (LMT, using “3”. Representative), 14 sets of high temperature overheating (HT, represented by “4”), and 26 sets of normal operation (N, represented by “5”). The states arrangement of samples is given in Table 2.

The data need to be preprocessed: Normalizing the data follows (19) to eliminate differences caused by ratio magnitude differences:

(19) $$x_{i.result}=\frac{x_{i}-x_{i.min}}{x_{i.max}-x_{i.min}}$$

where *x_i.result_* is the result of normalization, *x_i_* is the ratio which needs to be normalized, *x_i.max_* and *x_i.min_* are the maximum and the minimum members among entire samples.

### 4.2. Result of DGA Optimal Feature Selection

#### 4.2.1. Parameter Setting in Three Stage-GA–SA–SVM

In 3-stage GA–SA–SVM optimization, several parameters were specified in Table 3 and Table 4. The maximum iteration number was set at 200. The population scale was determined at 20. The number of chromosome segments was 3. Both *L*_1_ and *L*_2_ took 18, which guarantees that the upper bound of both *C* and *σ* is 255 and they can be accurate to 10^−4^. *L_3_* was 28. In the optimization process, the first 40 generations were fully optimized using the GA-SVM method, 40–180 generations utilized the GA–SA–SVM algorithm, and the Inverse SA–GA–SVM algorithm was applied in the last 20 generations.

#### 4.2.2. Comparison with Other Methods

**Figure 9:**
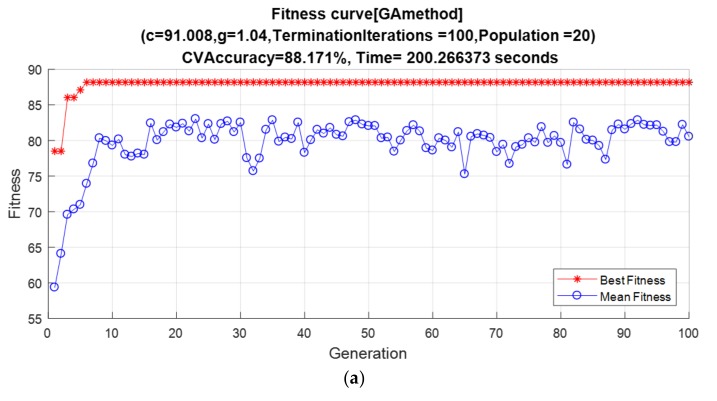

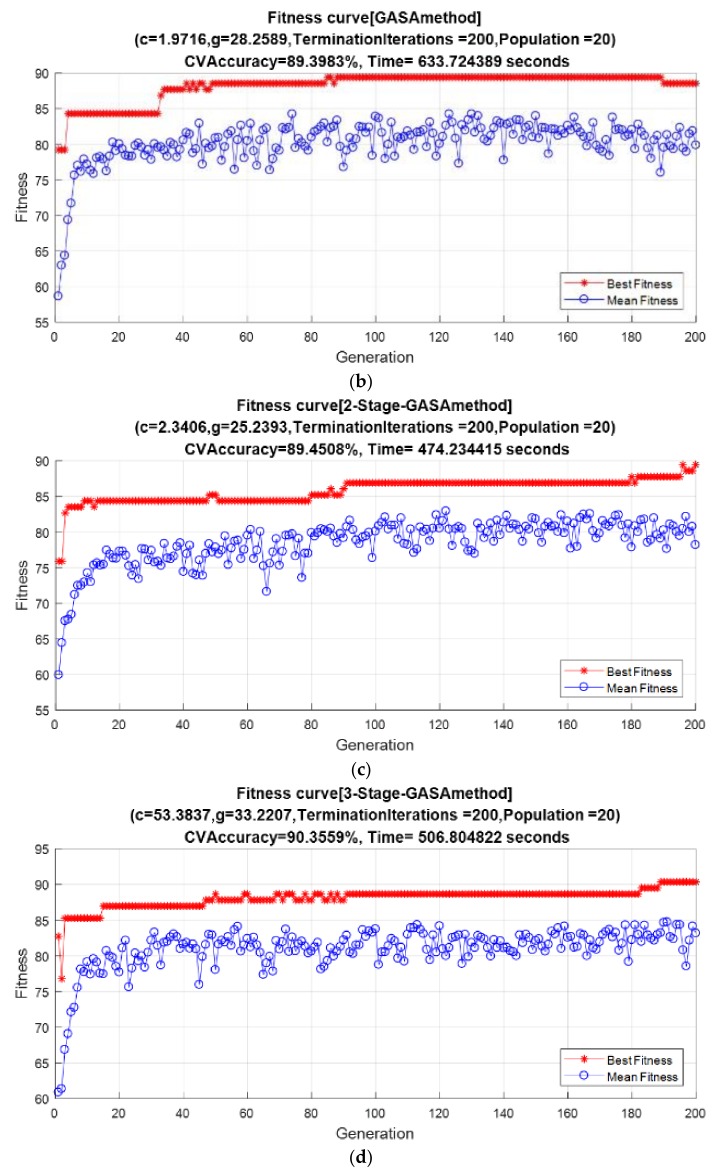
Results of four optimal feature selection methods: (**a**) The result of the GA–SVM method; (**b**) The result of the GA–SA–SVM method; (**c**) The result of the 2-stage GA–SA–SVM method; (**d**) The result of the 3-stage GA–SA–SVM method.

In order to embody the advantages of 3-stage GA–SA–SVM, we used GA–SVM, GA–SA–SVM, 2-stage GA–SA–SVM, and 3-stage GA–SA–SVM to select feature combinations and obtain the results of four methods. Each result includes CV accuracy, fitness curve, and optimal feature combination. CV accuracy and the optimal combination of features for each method are listed in Table 5. The fitness curve for each method is shown in Figure 9, which also included optimal *C* and *σ*. In the following figures, *g* is used to represent *σ* and *c* is used to represent *C*. In the meantime, we would like to emphasize that, in order to overcome the possible accuracy problems caused by insufficient public sample data of transformers, these results are carefully selected by the author after 50 times of repeated experiments, and are the closest to the average results.

In Table 5, 3-stage GA–SA–SVM has the highest CV accuracy among all algorithms. In Figure 9a, GA–SVM’s fitness reaches the highest value within 20 generations and takes the shortest time, which is only about 200 s, to end the iteration. However, due to the fitness curve no longer climbing after reaching a platform, the GA algorithm is more likely to be trapped in the local optimal solution, making the result unstable. Also, the accuracy of the GA–SVM is slightly lower than that of other algorithms. The GA–SA–SVM algorithm made some improvements based on the GA algorithm. In Figure 9b, the fitness of the GA–SA–SVM changed after arriving at a local optimization platform, which means that it is easier for the GA–SA–SVM to jump out of the local optimal solution. Therefore, the result of the GA–SA–SVM looks more stable and accurate. However, the weakness of the GA–SA–SVM is marked. The GA–SA–SVM’s fitness reaches the platform period at around the 40th generation and requires a long running time of more than 600 s. The adoption of the 2-stage GA–SA–SVM has already made some improvements to this problem. In Figure 9c, the 2-stage GA–SA–SVM was able to jump out of the local optimal solution and merely required 474 s to complete the optimization, and 20 generations to reach the local optimal platform. The shortcoming that remains in the 2-stage GA–SA–SVM is that the fitness may jump out of the global optimal solution at the end of the optimization. This situation is due to the temperature *T* in the SA algorithm is already very low within the last ten generations, and the probability of accepting a positive-direction-mutation (which makes the fitness grow) is quite low. In contrast, the probability of receiving a negative-direction-mutation is 100%. For example, maximum fitness in the 194th generation of Figure 9c decreased during accepting a negative-direction-mutation. This generation was very close to the maximum number of iterations. At this moment, it was a risk that the result may return to the local optimal solution and never grow again until the end of the iteration. This is a typical defect of the SA algorithm when the maximum number of iterations is set in the first place. A similar situation also occurs in the GA–SA–SVM hybrid algorithm: At the 190th generation in Figure 9b, fitness returns to the local optimal solution until the end of the iteration. The 3-stage GA–SA–SVM was intended to overcome this shortage. In Figure 9d, the 3-stage GA–SA–SVM retains all of the benefits of the 2-stage GA–SA–SVM, except that the solution takes a little longer—up to 506 s. After the 180th generation, the inverse SA algorithm not only eliminated the decrease in fitness that might occur in the SA algorithm, but also provided two opportunities for the fitness to raise. The 3-stage GA–SA–SVM is therefore more stable and accurate than the 2-stage GA–SA–SVM method.

Based on the high accuracy and stability of the 3-stage GA–SA–SVM, the selection results of the 3-stage GA–SA–SVM are considered to be the most reasonable OFC. DGA ratio components of the OFC are set out in Table 6.

### 4.3. ABC Diagnostic Results

#### 4.3.1. Parameter Setting in Three ABC–SVM

Based on the LpO CV, 118 sets of IEC TC 10 samples were divided into two groups. Among these, 93 sets were for training and 25 sets for testing. The states arrangement of testing samples is listed in Table 7. 

In the ABC algorithm, we set the scale of the bee colony to 20. The number of honey sources (solutions) is set to half of the scale, that is, 10. In each generation, the maximum number of extra honey sources that can be found are 100. That is, if the reconnaissance bees discover more than 100 fresh honey sources and the quality of the honey sources does not increase, reinitialize the honey sources. This setting is to prevent ABC from being trapped in the local optimal solution. The maximum number of loops is 10 and the dimension of the vector to be optimized is 2. The parameters are arranged as shown in Table 8.

#### 4.3.2. Diagnostic Result

**Figure 10:**
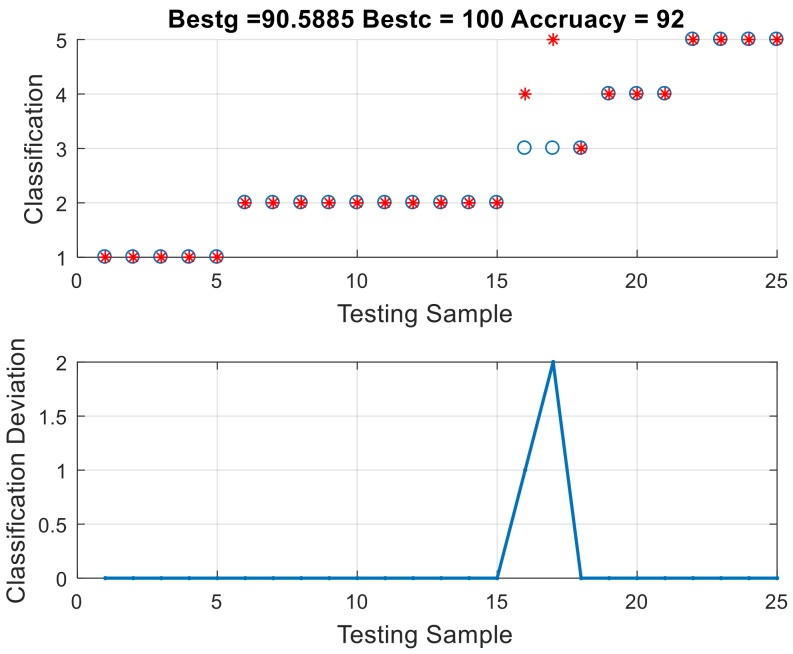
Diagnosis result of ABC–SVM.

The final results of the 25 testing sample diagnoses and the accuracy of the diagnosis are given in Figure 10. The blue circle in the upper half of the diagram represents the correct label status of the testing data, and the red dot is the diagnostic result from the diagnostic system. When the red dot coincides with the blue circle, it means that the result of diagnosis is accurate. The lower half figure depicts the distribution of the diagnostic deviation of the diagnostic system. Results showed that diagnostic accuracy amounts to 92% (23/25); errors only occurred at points 16 and 17. 

#### 4.3.3. Result of Comparison

We divided the comparison into two parts: Self-comparing and comparison with standard algorithms and other wrapper algorithms.

- Self-comparing

**Figure 11:**
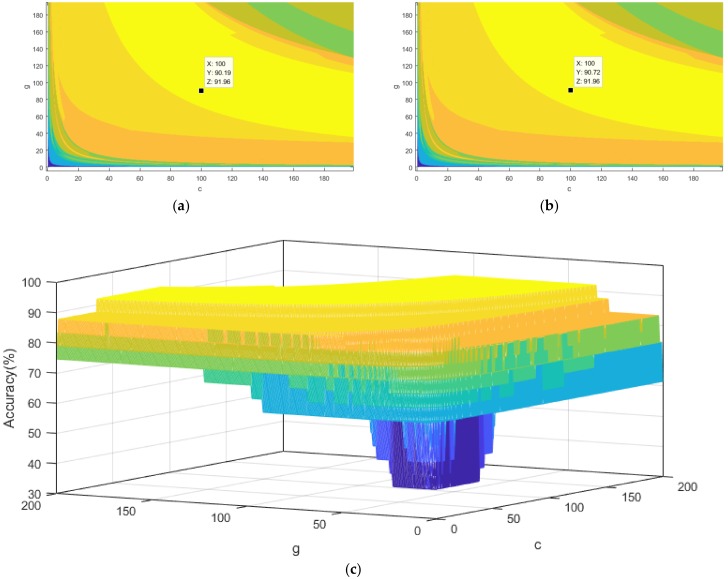
Testing accuracies for all the points (*c*, *σ*) (**a**) Cross-sections of the points at (100, 90.19); (**b**) Cross-sections of the points at (100, 90.72); (**c**) is a 3D visualization of all the points.

Since that the task of ABC algorithm is to search the optimal value of *C* and the *σ*, we simulated the diagnostic accuracy of each point of *C* and the *σ* to demonstrate the superiority of ABC. The *σ* and *C* are both on a [0, 200] × [0, 200] square plane. Their values correspond to the *X* axis and the *Y* axis on the plane, respectively. The Z axis perpendicular to the plane represents the accuracy of diagnosis. The detailed results of the simulation are presented in Figure 11, which depicted that the highest precision obtained by the SVM classifier was 91.96%. The diagnostic result of the ABC–SVM has reached the global highest point. The self-comparison verified that ABC has excellent performance under given data and operating conditions.

- Comparison with Standard Algorithms and other Wrapper Algorithms

**Figure 12:**
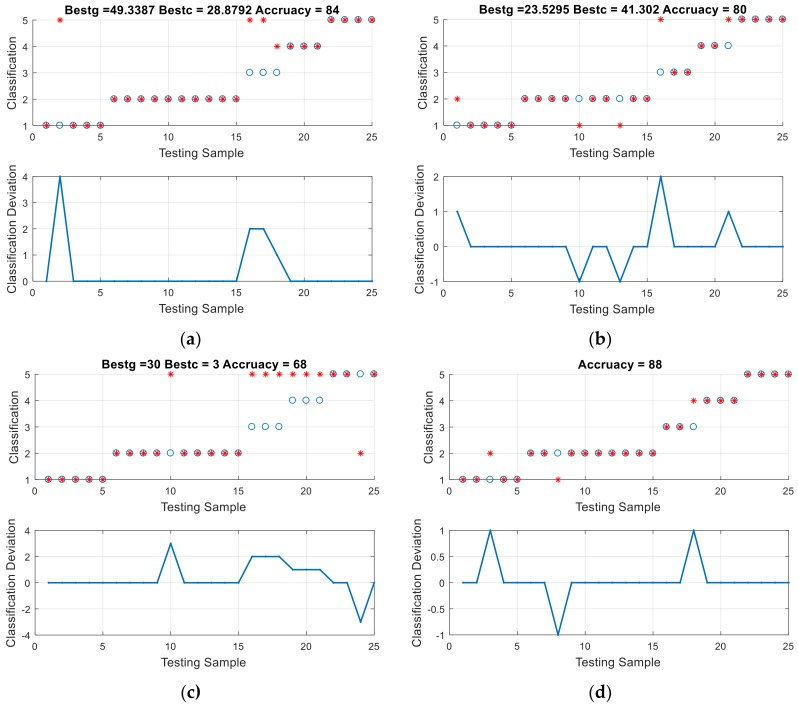
Fault diagnosis results and the spatial distribution of the optimal solution using different methods (**a**) PSO–SVM; (**b**) GA–SVM; (**c**) SVM; (**d**) BPNN.

**Figure 13:**
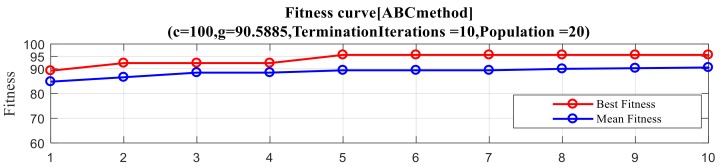

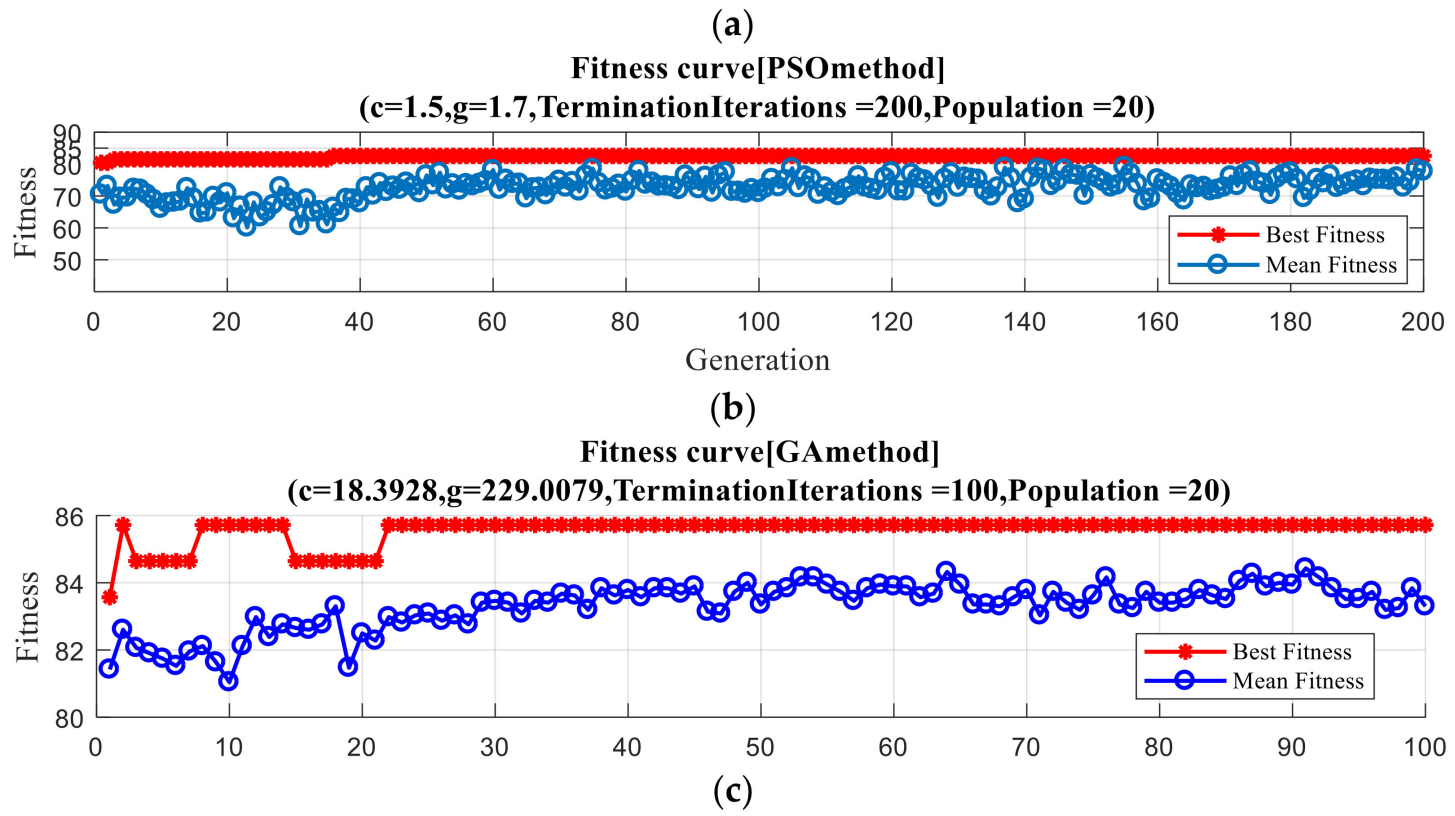
Average fitness and best fitness of SVM based methods (**a**) ABC method; (**b**) PSO method; (**c**) GA–SVM.

We compared the results obtained by ABC–SVM with the results originating from standard algorithms based methods: SVM, and back propagation neural networks (BPNN) and wrapper algorithms: GA–SVM and PSO–SVM. These methods shared the same sample, based on the optimal feature combination in Table 6. The diagnostic result of each method is listed in Figure 12. To compare with other wrapper algorithms, fitness curves of wrapper algorithms are shown in Figure 13. In the meantime, we would like to emphasize that, in order to overcome the possible accuracy problems caused by insufficient public sample data of transformers, these diagnostic results are carefully selected by the author after 50 times of repeated experiments, and are the closest to the average results.

The advantages of the ABC–SVM in terms of accuracy can be clearly seen from the comparison between Figure 10 and Figure 12. The ABC–SVM is the only one of all algorithms with a precision of over 90%. The diagnostic accuracy of ABC–SVM is obviously improved compared to that of standard SVM, and it is better than that of BPNN. The accuracy of ABC–SVM is also higher than that of other wrapper algorithms.

In addition, it can be seen that the ABC algorithm has better convergence characteristics when compared to other optimization algorithms, which guarantees that ABC–SVM performs better than other wrapper algorithms. It runs steadily, has fewer iterations, and has a rapid convergence time and a high termination fitness. Unlike GA or PSO, which require hundreds of generations of calculations, ABC reached the optimal platform within five iterations. Besides, as seen in Figure 13, termination fitness of ABC exceeded 95%, which is almost 10% higher than that of the GA and the PSO. This shows the ABC’s outstanding preferment in convergence.

## 5. Conclusions and Future Directions

This paper combines the traditional DGA method and intelligent algorithms and proposes a complete online monitoring and diagnostic system for power transformers. Inheriting the advantages of traditional DGA online technology, the novel diagnostic method has sound economic characteristics and alleviates the pressure brought by the allowable-time problem effectively. The diagnostic process includes: (1) An extracted DGA feature combination based on 3-stage GA–SA–SVM and (2) using the ABC–SVM classification model to diagnose transformer faults based on the optimal feature combination. The results are shown to be highly accurate and reliable. The system has strong anti-noise ability, so it requires less attention in the working environment condition.

In subsequent studies, we will concentrate on two research directions: (1) Developing an improved ABC algorithm to link SVM mode, and (2) designing an algorithm (ABC–SVR) that combines support vector regression (SVR) with ABC.

## Figures and Tables

**Table 1:** Dissolved gas analysis (DGA) Ratios.

Ratios	Ratios	Ratios	Ratios	Ratios
H_2_/CO	H_2_/CO_2_	H_2_/CH_4_	H_2_/C_2_H_2_	H_2_/C_2_H_4_
H_2_/C_2_H_6_	H_2_/TH	CO/CO_2_	CO/CH_4_	CO/C_2_H_2_
CO/C2H4	CO/C_2_H_6_	CO/TH	CO_2_/CH_4_	CO_2_/C_2_H_2_
CO_2_/C_2_H_4_	CO_2_/C_2_H_6_	CO_2_/TH	CH_4_/C_2_H_2_	CH_4_/C_2_H_4_
CH_4_/C_2_H_6_	CH_4_/TH	C_2_H_2_/C_2_H_4_	C_2_H_2_/C_2_H_6_	C_2_H_4_/TH
C_2_H_4_/C_2_H_6_	C_2_H_4_/TH	C_2_H_6_/TH	H_2_/C_2_H_2_	H_2_/C_2_H_4_

**Table 2:** The states arrangement of IEC TC 118.

Label	Quantity
1	23
2	45
3	10
4	14
5	26

**Table 3:** Parameters preset in 3-stage GA–SA–SVM.

Max Iteration	Population Scale	*L*_1_	*L*_2_	*L*_3_
200	20	22	22	28

**Table 4:** Generation interval of three stages.

GA–SVM	GA–SA–SVM	Inverse SA–GA–SVM
[0, 40]	[20, 180]	[180, 200]

**Table 5:** Result of DGA selections.

Method	CV Accuracy	Selected Combinations
GA–SVM	88.17%	H_2_/CO, H_2_/CO_2_, H_2_/TH, CO/CO_2_, CO/C_2_H_2_, CO_2_/CH_4_, CO_2_/C_2_H_4_, CH_4_/TH, C_2_H_4_/TH, C_2_H_4_/C_2_H_6_
GA–SA–SVM	89.40%	H_2_/CO, H_2_/CO_2_, H_2_/CH_4_, H_2_/C_2_H_2_, CO/CH_4_, CO/C_2_H_4_, CO/TH, CO_2_/CH_4_, CO_2_/C_2_H_4_, CO_2_/TH, C_2_H_2_/C_2_H_4_, C_2_H_2_/C_2_H_6_
2-stage GA–SA–SVM	89.45%	H_2_/CO, H_2_/C_2_H_2_, CO/CO_2_, CO/C_2_H_2_, CO_2_/CH_4_, CO_2_/C_2_H_4_, CH_4_/TH, C_2_H_4_/TH, C_2_H_4_/C_2_H_6_
3-stage GA–SA–SVM	90.36%	H_2_/CO_2_, H_2_/C_2_H_2_, H_2_/C_2_H_4_, H_2_/TH, CO_2_/CH_4_, CO_2_/C_2_H_2_, CO_2_/C_2_H_4_, CH_4_/C_2_H_6_, C_2_H_6_/TH, CH_4_/TH, C_2_H_2_/C_2_H_4_, C_2_H_2_/C_2_H_6_

**Table 6:** Optimal feature combination.

Ratios
H_2_/CO_2_, H_2_/C_2_H_2_, H_2_/C_2_H_4_, H_2_/TH, CO_2_/CH_4_, CO_2_/C_2_H_2_, CO_2_/C_2_H_4_, CH_4_/C_2_H_6_, C_2_H_6_/TH, CH_4_/TH, C_2_H_2_/C_2_H_4_, C_2_H_2_/C_2_H_6_

**Table 7:** The states arrangement of the testing sample.

Label	Quantity
1	5
2	10
3	3
4	3
5	4

**Table 8:** Parameters preset in ABC.

Number of Honey Source	Scale of The Bee Colony	Max Number of New Sources in One Generation	Max Number of Loops
10	20	100	10

## References

[1] Li J.S., Zhou H.W., Meng J., Yang Q., Chen B. (2018). Carbon emissions and their drivers for a typical urban economy from multiple perspectives: A case analysis for Beijing city. Appl. Energy.

[2] Liu J., Zheng H., Zhang Y., Zhou T., Zhao J., Li J., Liu J., Li J. (2018). Comparative Investigation on the Performance of Modified System Poles and Traditional System Poles Obtained from PDC Data for Diagnosing the Ageing Condition of Transformer Polymer Insulation Materials. Polymers.

[3] Zhang Y., Liu J., Zheng H., Wei H., Liao R., Sciubba E. (2017). Study on Quantitative Correlations between the Ageing Condition of Transformer Cellulose Insulation and the Large Time Constant Obtained from the Extended Debye Model. Energies.

[4] Liu J., Zheng H., Zhang Y., Wei H., Liao R. (2017). Grey Relational Analysis for Insulation Condition Assessment of Power Transformers Based Upon Conventional Dielectric Response Measurement. Energies.

[5] Borutzky W. (2011). Bond Graph Modelling of Engineering Systems.

[6] Djeziri M.A., Ananou B., Ouladsine M. Data driven and model based fault prognosis applied to a mechatronic system. Proceedings of the Fourth International Conference on Power Engineering, Energy and Electrical Drives.

[7] Sun H.-C., Huang Y.-C., Huang C.-M. (2012). A Review of Dissolved Gas Analysis in Power Transformers. Energy Procedia.

[8] Sica F.C., Guimarães F.G., Duarte R.d.O., Reis A.J.R. (2015). A cognitive system for fault prognosis in power transformers. Electr. Power Syst. Res..

[9] Bengtsson C. (1996). Status and trends in transformer monitoring. IEEE Trans. Power Deliv..

[10] Yang M.-T., Hu L.-S. (2013). Intelligent fault types diagnostic system for dissolved gas analysis of oil-immersed power transformer. IEEE Trans. Dielectr. Electr. Insul..

[11] Singh S., Bandyopadhyay M.N. (2010). Dissolved gas analysis technique for incipient fault diagnosis in power transformers: A bibliographic survey. IEEE Electr. Insul. Mag..

[12] Duval M., Pablo A.D., Atanasova-Hoehlein I., Grisaru M. (2017). Significance and detection of very low degree of polymerization of paper in transformers. IEEE Electr. Insul. Mag..

[13] Peischl S., Walker J.P., Ryu D., Kerr Y.H. (2017). Analysis of Data Acquisition Time on Soil Moisture Retrieval from Multiangle L-Band Observations. IEEE Trans. Geosci. Remote.

[14] Unsworth J., Mitchell F. (1990). Degradation of electrical insulating paper monitored with high performance liquid chromatography. IEEE Trans. Electr. Insul..

[15] Verma H.C., Baral A., Pradhan A.K., Chakravorti S. (2017). A method to estimate activation energy of power transformer insulation using time domain spectroscopy data. IEEE Trans. Dielectr. Electr. Insul..

[16] Saha T.K., Purkait P., Muller F. An attempt to correlate time & frequency domain polarisation measurements for the insulation diagnosis of power transformer. Proceedings of the Power Engineering Soc. General Meeting.

[17] Bakar N.A., Abu-Siada A., Islam S. (2014). A review of dissolved gas analysis measurement and interpretation techniques. IEEE Electr. Insul. Mag..

[18] Gómez N.A., Wilhelm H.M., Santos C.C., Stocco G.B. (2014). Dissolved gas analysis (DGA) of natural ester insulating fluids with different chemical compositions. IEEE Trans. Dielectr. Electr. Insul..

[19] Rogers R.R. (1978). IEEE and IEC Codes to Interpret Incipient Faults in Transformers, Using Gas in Oil Analysis. IEEE Trans. Electr. Insul..

[20] Duval M., Depabla A. (2001). Interpretation of gas-in-oil analysis using new IEC publication 60599 and IEC TC 10 databases. IEEE Electr. Insul. Mag..

[21] Irungu G.K., Akumu A.O., Munda J.L. (2016). A new fault diagnostic technique in oil-filled electrical equipment; the dual of Duval triangle. IEEE Trans. Dielectr. Electr. Insul..

[22] Irungu G.K., Akumu A.O., Munda J.L. Comparison of IEC 60599 gas ratios and an integrated fuzzy-evidential reasoning approach in fault identification using dissolved gas analysis. Proceedings of the International Universities Power Engineering Conference (UPEC).

[23] Barbosa T.M., Ferreira J.G., Finocchio M.A.F., Endo W. (2017). Development of an Application Based on the Duval Triangle Method. IEEE Latin Am. Trans..

[24] Benmahamed Y., Teguar M., Boubakeur A. (2017). Application of SVM and KNN to Duval Pentagon 1 for transformer oil diagnosis. IEEE Trans. Dielectr. Electr. Insul..

[25] Faiz J., Soleimani M. (2017). Dissolved gas analysis evaluation in electric power transformers using conventional methods a review. IEEE Trans. Dielectr. Electr. Insul..

[26] Islam S.M., Wu T., Ledwich G. (2000). A novel fuzzy logic approach to transformer fault diagnosis. IEEE Trans. Dielectr. Electr. Insul..

[27] Miranda V., Castro A.R.G. (2005). Improving the IEC table for transformer failure diagnosis with knowledge extraction from neural networks. IEEE Trans. Power Deliv..

[28] Zheng H., Zhang Y., Liu J., Wei H., Zhao J., Liao R. (2018). A novel model based on wavelet LS-SVM integrated improved PSO algorithm for forecasting of dissolved gas contents in power transformers. Electr. Power Syst. Res..

[29] Zhang Y., Wei H., Liao R., Wang Y., Yang L., Yan C. (2017). A New Support Vector Machine Model Based on Improved Imperialist Competitive Algorithm for Fault Diagnosis of Oil-immersed Transformers. J. Electr. Eng. Technol..

[30] Dai J., Song H., Sheng G., Jiang X. (2017). Dissolved gas analysis of insulating oil for power transformer fault diagnosis with deep belief network. IEEE Trans. Dielectr. Electr. Insul..

[31] Mirowski P., LeCun Y. (2012). Statistical Machine Learning and Dissolved Gas Analysis: A Review. IEEE Trans. Power Deliv..

[32] Huang Y.C. (2003). A new data mining approach to dissolved gas analysis of oil-insulated power apparatus. IEEE Trans. Power Deliv..

[33] Chen W., Pan C., Yun Y., Liu Y. (2008). Wavelet Networks in Power Transformers Diagnosis Using Dissolved Gas Analysis. IEEE Trans. Power Deliv..

[34] Zhou J., Yang Y., Ding S.X, Zi Y., Wei M. (2018). A Fault Detection and Health Monitoring Scheme for Ship Propulsion Systems Using SVM Technique. IEEE Access.

[35] Zhang Y., Zheng H., Liu J., Zhao J., Sun P. (2018). An Anomaly Identification Model for Wind Turbine State Parameters. J. Clean. Prod..

[36] Qin L., Wang J., Li H., Sun Y., Li S. (2017). An Approach to Improve the Performance of Simulated Annealing Algorithm Utilizing the Variable Universe Adaptive Fuzzy Logic System. IEEE Access.

[37] Xin F., Ni S., Li H., Zhou X. (2018). General Regression Neural Network and Artificial-Bee-Colony Based General Regression Neural Network Approaches to the Number of End-of-Life Vehicles in China. IEEE Access.

[38] Tang W.H., Goulermas J.Y., Wu Q.H., Richardson Z.J., Fitch J. (2008). A Probabilistic Classifier for Transformer Dissolved Gas Analysis with a Particle Swarm Optimizer. IEEE Trans. Power Deliv..

[39] Kim S.W., Kim S.J., Seo H.D., Jung J.R., Yang H.J., Duval M. (2013). New methods of DGA diagnosis using IEC TC 10 and related databases Part 1: Application of gas-ratio combinations. IEEE Trans. Dielectr. Electr. Insul..

[40] Karaboga D., Akay B. (2009). A survey: Algorithms simulating bee swarm intelligence. Artif. Intell. Rev..

